# Association of Carotid Plaque Gray-scale Median with Plaque Size and Degree of Stenosis

**DOI:** 10.2174/0115734056424582260218072204

**Published:** 2026-03-16

**Authors:** Salahaden R Sultan

**Affiliations:** 1 Department of Radiologic Sciences, Faculty of Applied Medical Sciences, King Abdulaziz University, Jeddah, Saudi Arabia; 2 Department of Radiology, King Abdulaziz University Hospital, King Abdulaziz University, Jeddah, Saudi Arabia

**Keywords:** B-mode, Ultrasound, Atherosclerosis, Carotid plaque, Echogenicity, Gray-scale median

## Abstract

**Introduction:**

Atherosclerosis remains a leading cause of Cardiovascular Disease (CVD) mortality and morbidity. Ultrasound Gray-Scale Median (GSM) of carotid plaques and plaque size have shown potential as markers of vulnerability and predictors of cerebrovascular events. This prospective study investigated the correlation between carotid plaque size, Degree of Stenosis (DoS), and GSM.

**Materials and Methods:**

In this study, 32 patients with 43 carotid plaques identified by B-mode ultrasound were recruited. Each B-mode ultrasound image was normalized, and the GSM of carotid plaque echogenicity was quantified using the histogram feature in Adobe Photoshop. GSM data from the thickest plaque area were obtained, and plaque length, thickness, area, and DoS were measured using ImageJ software. Correlations between plaque size, DoS, and GSM were identified and assessed using Spearman’s correlation coefficient.

**Results:**

Significant negative correlations were observed between carotid plaque GSM and plaque size (plaque length, r = −0.34, *p* = 0.02; plaque thickness, r = −0.30, *p* = 0.04; plaque area, r = −0.40, *p* = 0.008), with a trend toward significant negative correlation with DoS (r = −0.26, *p* = 0.08).

**Discussion:**

This study identifies an inverse relationship between carotid plaque GSM and plaque size parameters, indicating that hypoechoic plaques, characterized by lower GSM values, are generally larger and potentially more vulnerable to rupture. These findings emphasize the importance of combining structural metrics, such as plaque size and degree of stenosis, with compositional evaluation and their echogenicity for comprehensive cerebrovascular risk assessment. Hypoechoic plaques often correspond to lipid-rich, inflamed lesions with thin fibrous caps, which are histological features associated with an increased risk of cerebrovascular events. The demonstrated associations underscore the clinical relevance of GSM as a noninvasive surrogate biomarker that reflects both local plaque vulnerability and the broader systemic burden of atherosclerosis. Thus, the B-mode ultrasound-derived GSM is a practical and informative tool for enhancing risk stratification and guiding management strategies in patients with carotid artery disease.

**Conclusion:**

Our findings reveal significant negative correlations between carotid GSM and plaque size parameters, with a nonsignificant trend for DoS. These findings suggest that hypoechoic plaques, characterized by lower GSM values, are generally larger in size and potentially more vulnerable. Future large-scale longitudinal studies with repeated evaluations are required to validate these findings.

## 1 INTRODUCTION

Atherosclerosis, a chronic inflammatory disease characterized by the accumulation of lipids, inflammatory cells, and fibrous elements in the arterial wall, is a leading cause of Cardiovascular Disease (CVD)-associated morbidity and mortality, posing significant global health challenges and socioeconomic burdens [1]. The progressive narrowing and hardening of arteries caused by plaque accumulation predispose individuals to ischemic events and pose substantial challenges for healthcare systems [2]. Understanding the structural and compositional characteristics of these plaques, including their morphology, is crucial for predicting cardiovascular and cerebrovascular events and optimizing clinical interventions [3].

Atherosclerotic plaques are a significant risk factor for adverse cardiovascular and cerebrovascular events, including stroke and myocardial infarction, which are leading global causes of disability and mortality [4, 5]. The progression and clinical consequences of carotid artery disease are influenced by the structural and compositional characteristics of atherosclerotic plaques within the vessel wall [6]. A comprehensive evaluation of carotid plaque characteristics is crucial for risk stratification and guiding treatment decisions in patients with carotid artery disease [7]. The evaluation of carotid plaques has traditionally centered on luminal stenosis as a key parameter [8]. In symptomatic patients, stroke risk is closely associated with increasing stenosis severity; however, the correlation between stenosis severity and stroke risk becomes less distinct in asymptomatic individuals with stenosis exceeding 50–60%, indicating that additional factors may substantially contribute to stroke risk [8, 9]. Although the Degree of Stenosis (DoS) is an important indicator for cardiovascular risk stratification, recent evidence highlights the critical roles of plaque composition and morphology in determining plaque vulnerability [10, 11].

Ultrasound imaging has emerged as a valuable, noninvasive tool for evaluating carotid atherosclerosis. In particular, B-mode ultrasound enables the visualization of carotid plaque morphology and the quantification of plaque size and DoS [12]. The Gray-Scale Median (GSM) obtained from B-mode ultrasound is a reliable quantitative tool for assessing plaque echogenicity, enabling differentiation between symptomatic and asymptomatic plaques and providing valuable insights into plaque morphology [13]. Studies have revealed a correlation between GSM values and plaque histopathology, with lower GSM values indicating lipid-rich, inflamed, and vulnerable hypoechoic plaques, and higher GSM values reflecting calcified, fibrotic, and more stable hyperechoic plaques, highlighting the clinical potential of the GSM in identifying vulnerable plaques and assessing ischemic risk [14-17]. Larger carotid plaques are considered more vulnerable because their increased surface area heightens the risk of flow obstruction and cerebrovascular events, with plaque size strongly linked to cerebral symptoms and both plaque length and area correlating with microemboli and ischemic stroke [12, 18-20]. Studies have also demonstrated that both the size and echogenicity of carotid plaques, as assessed through ultrasound, are independent predictors of future cerebrovascular events [19, 21]. However, the implications of carotid plaquecompositiononplaquesize and DoS require investigation. Therefore, this study was conducted to investigate the relationship between carotid plaque size and plaque composition obtained from the GSM assessed *via* ultrasound in a cohort of patients with carotid plaques.

## 2 MATERIALS AND METHODS

### 2.1 Study Design and Data Acquisition

This prospective, observational pilot study analyzed data derived from carotid ultrasound examinations conducted on adult patients diagnosed with carotid plaques at King Abdullah Medical City, Makkah, Saudi Arabia. Patients who underwent carotid ultrasound examination with no carotid plaques observed on B-mode images were excluded. Ethical approval was obtained (IRB No. 23-1177). The study design adhered to the principles outlined in the Declaration of Helsinki and the International Conference on Harmonization of Good Clinical Practice. Patients referred to the ultrasound department for carotid examination between March and December 2024 were recruited, and written informed consent was obtained. Patients with plaques obscured by acoustic shadowing and those unable to give informed consent were excluded.

**Fig. (1):**
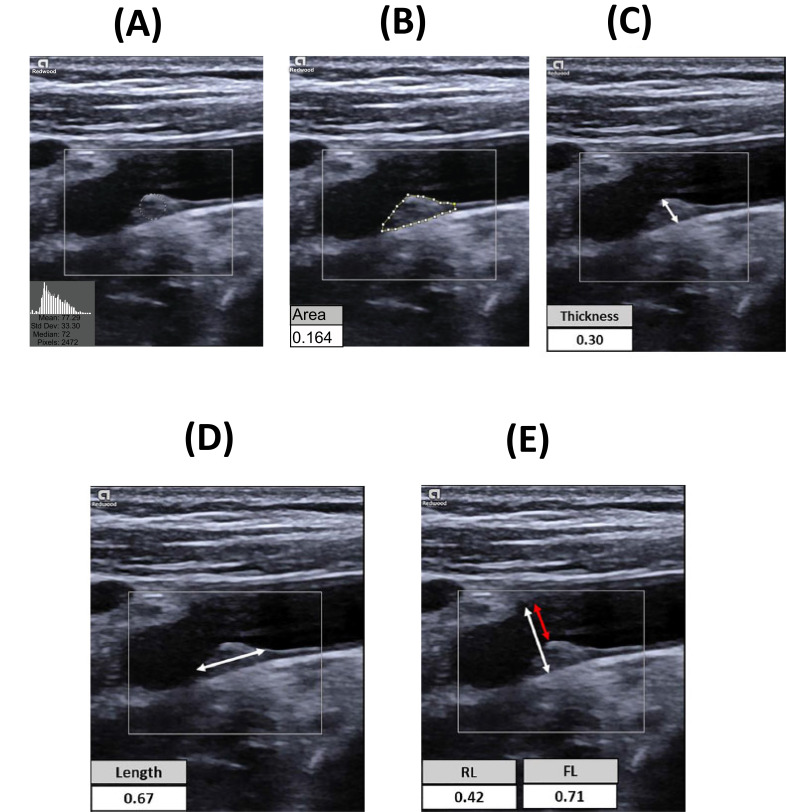
Measurements of carotid plaque gray-scale median (**A**), area (**B**), thickness (**C**), length (**D**), and residual and full lumen diameters (**E**). A. Gray-Scale Median (GSM) measurement for the selected region of interest within the plaque. B. Measurement of the plaque area. C. Measurement of plaque thickness. D. Measurement of plaque length. E. Measurement of the full lumen length (FL, white arrow) and residual lumen diameter (RL, red arrow) for calculating the degree of stenosis.

Demographic and clinical information, encompassing age, sex, hypertension, diabetes, and smoking status, was collected. Carotid ultrasound assessments were conducted by an experienced clinical vascular scientist using a high-resolution ACUSON Redwood ultrasound imaging system (Siemens Healthineers, Erlangen, Germany). The GSM was determined by normalizing B-mode ultrasound images using automatic linear scaling, setting blood intensity to 0 and adventitia to 255, followed by calculating plaque echogenicity using the histogram analysis feature in Adobe Photoshop [16, 18]. GSM values from the thickest plaque area were obtained. Plaque length, thickness, area, and DoS were measured using ImageJ software. The DoS was calculated using the standard formula of the European Carotid Surgery Trial, as follows: DoS ​(%)= [1 − (residual lumen at the most stenotic point / full lumen diameter)] × 100 [22] (Fig. **1A-E**).

### 2.2 Statistical Analysis

The association between the carotid plaque size, DoS, and GSM was assessed using Spearman’s rank correlation analysis. The level of significance was set at *p* < 0.05. Blinded data analysis was performed using IBM SPSS Statistics version 21 (Armonk, NY: IBM Corp) and GraphPad PRISM 7 (La Jolla, CA, USA).

## 3 RESULTS

**Fig. (2):**
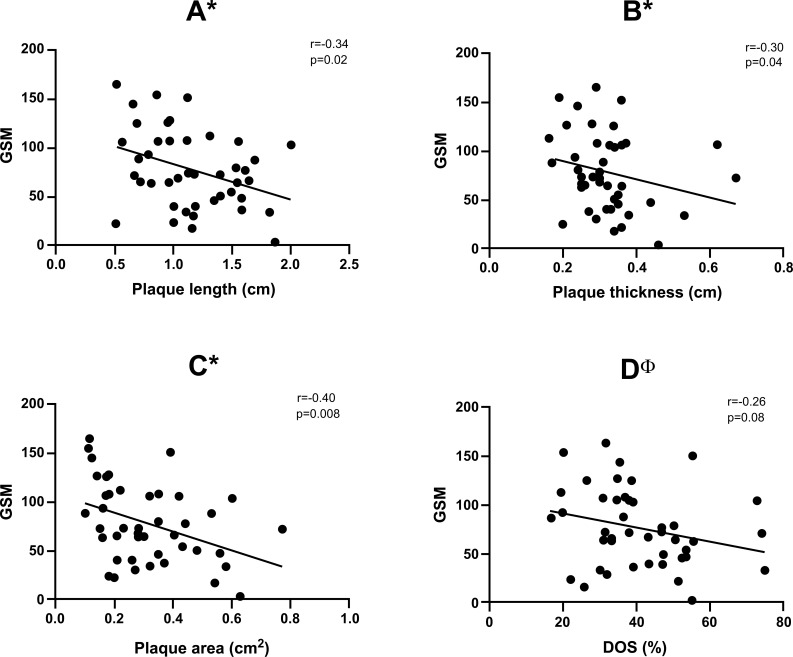
Correlation of the Gray-Scale Median (GSM) with plaque length, size, and degree of stenosis. (**A**). Correlation between GSM (x-axis) and plaque length (y-axis, cm). (**B**). Correlation between GSM (x-axis) and plaque thickness (y-axis, cm). (**C**). Correlation between GSM (x-axis) and plaque area (y-axis, cm^2^). (**D**). Correlation between the GSM (x-axis) and degree of stenosis (y-axis, DoS, %). *p ≤ 0.05 and ^ɸ^*p* ≤ 0.09 using Spearman’s rank correlation.

Ultrasound B-mode images of 43 carotid plaques from 32 patients were evaluated. Twenty-one of the patients were male, 12 had cerebrovascular symptoms, 25 had hypertension, 24 were diabetic, and 11 were smokers. The average GSM was 77.33 ± 39.92. The average DoS and plaque sizes were as follows: DoS, 40.75 ± 14.13%; plaque length, 1.15 ± 0.38 cm; plaque thickness, 0.32 ± 0.10 cm; and area, 0.31 ± 0.16 cm^2^. The patient information is summarized in Table **1**. Significant negative correlations were observed between the carotid plaque GSM and plaque size (plaque length, r = −0.34, *p* = 0.02; plaque thickness, r= −0.30, *p* = 0.04; plaque area, r = −0.40, *p* = 0.008, (Fig. **2A**-**C**), with a trend toward a significant negative correlation with DoS (r= −0.26, *p* = 0.08, Fig. **2D**).

## 4 DISCUSSION

This study highlights the relationship between carotid plaque characteristics and their vulnerability, providing valuable insights for risk stratification in carotid artery disease. Significant negative correlations were determined between the carotid plaque GSM and plaque size metrics, including length, thickness, and area. A trend toward significance was observed between the GSM and DoS. These findings suggest that plaques with lower GSM values tend to be larger in size, underscoring the importance of evaluating plaque composition and echogenicity in addition to stenosis severity. These observations, integrating structural and compositional assessments, emphasize the need for a comprehensive approach to cerebrovascular risk evaluation and management.

Various risk factors, including aging, cardiovascular risk factors, markers of inflammation, and oxidation, have been reported to be associated with plaque size and echogenicity [22, 23]. It has been reported that aging, male sex, arterial hypertension, diabetes mellitus, hypercholesterolemia, and smoking influence carotid plaque progression [23, 24]. Additionally, the migration of circulating monocytes into the vessel wall is a key event in the initiation of atherosclerotic plaque formation, which is mediated by the expression of adhesion molecules in response to endothelial stimulation or damage, caused by arterial hypertension, turbulent blood flow, and smoking [24].

Inflammatory and lipid contents in carotid plaques are crucial factors that influence carotid plaque size. The composition of carotid plaques, particularly lipid content, is strongly associated with systemic inflammation, and the presence of atherosclerotic disease in carotid arteries is a substantial risk for cerebrovascular events [24]. Carotid plaque lipid content and fibrous cap status are closely related to the risk of cerebrovascular events, indicating that plaque composition is more important than plaque size in predicting cerebrovascular events [25]. Lipid profiles, including higher LDL-C and lower HDL-C levels, are associated with an increased risk of carotid plaque and atherosclerosis, suggesting a correlation between these lipid profiles, carotid plaque composition, and cardiovascular disease [26, 27]. Associations have also been shown between traditional cardiovascular risk factors, *i.e.*, markers of inflammation and oxidation, and plaque size and echogenicity, specifically linking increased Framingham risk scores, systolic blood pressure, and BMI, and decreased HDL and glutathione levels with echolucent plaques. Smoking is significantly associated with echorich plaques, with plaque size correlating with increased Framingham risk scores, systolic blood pressure, blood glucose levels, smoking levels, ApoB/A1 ratios, OxLDL levels, TNF alpha levels, HOMA insulin resistance, leukocyte counts, and decreased BCD-LDL and l-selectin levels [28, 29]. Taken together, these findings highlight the relationship between carotid plaque size and the GSM obtained from B-mode ultrasound.

## 5 STUDY LIMITATIONS

Carotid plaques were analyzed using data from a single visit, which may have been insufficient to accurately capture dynamic changes in plaque echogenicity over time. Longitudinal studies incorporating repeated ultrasound evaluations are essential for determining the temporal relationship between the GSM and plaque size. Furthermore, GSM measurements were obtained from the thickest plaque area to standardize measurements and minimize variability associated with image selection. However, because plaques exhibit considerable spatial heterogeneity, multiple region-of-interest sampling can facilitate a more comprehensive assessment of plaque echogenicity. Additionally, this study did not control for potential confounding variables, such as patient demographics, medical history, and medication use, which could influence the observed associations. Notably, a recent investigation identified factors such as elevated D-dimer levels, fasting plasma glucose, hypertension severity, and statin therapy as significant determinants of the GSM [18]. These findings highlight the complex interplay among clinical variables in determining plaque echogenicity. These factors should be considered in future studies to generate a more accurate estimate of the association. Finally, this study had a relatively small sample size; larger studies with more diverse patient populations are needed to validate these findings and further clarify the role of the GSM in assessing symptomatic and asymptomatic carotid plaque.

## CONCLUSION

This study reports a significant inverse relationship between the carotid plaque GSM and plaque size, and a nonsignificant trend observed for DoS. These findings indicate that plaques with lower GSM values are generally larger in size, supporting their potential association with increased plaque vulnerability. Further large-scale longitudinal studies are needed to confirm and expand these observations.

## Figures and Tables

**Table 1:** Patient and carotid plaque descriptive information.

**Patient, *n* [%]**	32 [100]
**Male, *n* [%]**	21 [65.63]
**Age, mean±SD [years]**	67.65±8.89
**Cerebrovascular symptoms**	12 [37.5]
**Hypertension, *n* [%]**	25 [78.13]
**Diabetes, *n* [%]**	24 [75.00]
**Smoking, *n* [%]**	11 [34.38]
**Plaque, *n* [%]**	43 [100]
**GSM, mean±SD**	77.33±39.92
**DoS, mean±SD [%]**	40.75±14.13
**Plaque length, mean±SD [cm]**	1.15±0.38
**Plaque thickness, mean±SD [cm]**	0.32±0.10
**Plaque area, mean±SD [cm^2^]**	0.31±0.16

**Abbreviations:** DoS: degree of stenosis, GSM: gray-scale median, *n*: number, SD: standard deviation.

## Data Availability

The authors confirm that the data supporting the findings of this research are available within the article.

## LIST OF ABBREVIATIONS

CVD: Cardiovascular Disease
GSM: Gray-Scale Median
DoS: Degree of Stenosis

## STANDARDS OF REPORTING

STROBE guidelines were followed.

## References

[1] Vasan R.S., Pan S., Larson M.G., Mitchell G.F., Xanthakis V. (2021). Arteriosclerosis, Atherosclerosis, and Cardiovascular Health: Joint Relations to the Incidence of Cardiovascular Disease.. Hypertension.

[2] Tsao C.W., Aday A.W., Almarzooq Z.I., Alonso A., Beaton A.Z., Bittencourt M.S., Boehme A.K., Buxton A.E., Carson A.P., Commodore-Mensah Y., Elkind M.S.V., Evenson K.R., Eze-Nliam C., Ferguson J.F., Generoso G., Ho J.E., Kalani R., Khan S.S., Kissela B.M., Knutson K.L., Levine D.A., Lewis T.T., Liu J., Loop M.S., Ma J., Mussolino M.E., Navaneethan S.D., Perak A.M., Poudel R., Rezk-Hanna M., Roth G.A., Schroeder E.B., Shah S.H., Thacker E.L., VanWagner L.B., Virani S.S., Voecks J.H., Wang N.Y., Yaffe K., Martin S.S. (2022). Heart Disease and Stroke Statistics-2022 Update: A Report From the American Heart Association.. Circulation.

[3] Libby P. (2021). The changing landscape of atherosclerosis.. Nature.

[4] Feigin V.L., Abate M.D., Abate Y.H., Abd ElHafeez S., Abd-Allah F., Abdelalim A., Abdelkader A., Abdelmasseh M., Abd-Elsalam S., Abdi P., Abdollahi A., Abdoun M., Abd-Rabu R., Abdulah D.M., Abdullahi A., Abebe M., Abeldaño Zuñiga R.A., Abhilash E.S., Abiodun O.O., Abiodun O., Abo Kasem R., Aboagye R.G., Abouzid M., Abreu L.G., Abrha W.A., Abtahi D., Abu Rumeileh S., Abualhasan A., Abualruz H., Abu-Gharbieh E., Abukhadijah H.J., Abu-Rmeileh N.M.E., Aburuz S., Abu-Zaid A., Acuna J.M., Adane D.E., Adane M.M., Addo I.Y., Adedoyin R.A., Adegboye O.A., Adekanmbi V., Adhikari K., Adnani Q.E.S., Adra S., Adzigbli L.A., Afify A.Y., Afolabi A.A., Afrashteh F., Afzal M.S., Afzal S., Aghamiri S., Agyemang-Duah W., Ahinkorah B.O., Ahmad A., Ahmad M.M., Ahmad S., Ahmad S., Ahmad T., Ahmadzade A.M., Ahmed A., Ahmed A., Ahmed H., Ahmed S.A., Ajami M., Aji B., Akara E.M., Akinyemi R.O., Akkaif M.A., Akrami A.E., Al Awaidy S., Al Hamad H., Al Hasan S.M., Al Qadire M., Al Ta’ani O., Al-Ajlouni Y., Alalalmeh S.O., Alalwan T.A., Al-Aly Z., Al-amer R.M., Aldhaleei W.A., Aldossary M.S., Alemohammad S.Y., Al-Fatly B., Al-Gheethi A.A.S., Alhalaiqa F.N., Alharrasi M., Ali A., Ali M.U., Ali R., Ali S.S., Ali W., Al-Ibraheem A., Alif S.M., Aljunid S.M., Almahmeed W., Al-Marwani S., Alomari M.A., Alonso J., Alqahtani J.S., Al-Raddadi R.M.M., Alrawashdeh A., Alsabri M.A., Alshahrani N.Z., Altaany Z., Altaf A., Al-Tammemi A.B., Altwalbeh D., Alvis-Guzman N., Alwafi H., Al-Wardat M., Al-Worafi Y.M., Aly H., Aly S., Alyahya M.S.I., Alzoubi K.H., Al-Zyoud W.A., Amani R., Amegbor P.M., Amera T.G., Amin T.T., Amindarolzarbi A., Amiri S., Amu H., Amugsi D.A., Amusa G.A., Ancuceanu R., Anderlini D., Angappan D., Anil A., Ansari M.T.T., Ansari-Moghaddam A., Ansong R., Anvari S., Anwar S., Anwar S.L., Anyabolo E.E., Anyasodor A.E., Apostol G.L.C., Appiah F., Aqeel M., Arabloo J., Arabzadeh Bahri R., Arafat M., Aravkin A.Y., Ardekani A., Areda D., Aregawi B.B., Aregu G.M., Aremu O., Arifin H., Ärnlöv J., Artamonov A.A., Arulappan J., Aryal U.R., Aryan Z., Asbeutah A.M., Asemahagn M.A., Asemu M.T., Asghari-Jafarabadi M., Ashemo M.Y., Ashraf T., Aslani A., Asmerom H.A., Astell-Burt T., Athari S.S., Atorkey P., Atout M.M.W., Atreya A., Aujayeb A., Ausloos M., Avan A., Awad H., Awotidebe A.W., Ayana L.A.A., Aychiluhm S.B., Aynalem A.A., Aynalem Z.B., Azadnajafabad S., Azami H., Aziz S., Azzam A.Y., Babu A.S., Babu G.R., Badar M., Badiye A.D., Bahrami Taghanaki P., Bahramian S., Bai R., Baig A.A., Bakkannavar S.M., Bako A.T., Baltatu O.C., Bam K., Banach M., Banakar M., Bandyopadhyay S., Banik P.C., Bansal K., Bao Y., Barboza M.A., Bardhan M., Barengo N.C., Barker-Collo S.L., Bärnighausen T.W., Barqawi H.J., Barrow A., Barua L., Bashiri A., Bashiru H.A., Basiru A., Bastan M-M., Basu S., Basu S., Batra K., Begde A., Behnam B., Behnoush A.H., Belayneh M.B.Y., Belingheri M., Bello U.M., Bennett D.A., Bensenor I.M., Berhe F.T., Bermudez A.N.C., Beyene H.B.B., Beyene K.A., Bhagat D.S., Bhagavathula A.S., Bhala N., Bhalla A., Bhardwaj N., Bhardwaj P., Bhaskar S., Bhat A.N., Bhat V., Bhatti G.K., Bhatti J.S.S., Bhuiyan M.A., Bhusal S., Bikbov B., Bilgin C., Biondi A., Bishaw K.A., Biswas A., Biswas B., Bodhare T., Bogale E.K., Boloor A., Bonakdar Hashemi M., Bonny A., Bora Basara B., Borhany H., Bosoka S.A., Bouaoud S., Bouyahya A., Boyko E.J., Bozic M.M., Braithwaite D., Breitner S., Brenner H., Britton G., Brunoni A.R., Bryazka D., Bugiardini R., Bulto L.N., Burkart K., Bustanji Y., Butt Z.A., Caetano dos Santos F.L., Cámera L.A., Campos L.A., Campos-Nonato I.R., Cao F., Capodici A., Cárdenas R., Carr S., Carreras G., Carvalho A.F., Carvalho F., Castaldelli-Maia J.M., Castañeda-Orjuela C.A., Castelpietra G., Catapano A.L., Cattaruzza M.S., Cegolon L., Cembranel F., Cenko E., Cerin E., Chadwick J., Chakraborty C., Chakraborty S., Chan J.S.K., Chandika R.M., Chandrasekar E.K., Chanie G.S., Chattu V.K., Chaudhary A.A., Chaurasia A., Chen H., Chen M., Chen S., Chi G., Chichagi F., Chimoriya R., Ching P.R., Chitheer A., Cho S.M.J., Choi D-W., Chong B., Chong C.L., Chopra H., Choudhari S.G., Choudhary R., Chu D-T., Chukwu I.S., Chung S-C., Cindi Z., Cioffi I., Cogen R.M., Columbus A., Costanzo S., Couto R.A.S., Criqui M.H., Cruz-Martins N., Cuadra-Hernández S.M., da Silva A.G., Dadana S., Dadras O., Dai X., Dalal K., Dalli L.L., Damiani G., D’Amico E., Dandona L., Dandona R., Darwish A.H., Das S., Dashti M., Dashtkoohi M., Dashtkoohi M., Dastmardi M., Davletov K., De la Cruz-Góngora V., DeAngelo S., Debele A.T., Debopadhaya S., Delgado-Enciso I., Demessa B.H., Demetriades A.K., Denova-Gutiérrez E., Dervišević E., Desai H.D., Desale A.T., Desta F., Devanbu V.G.C., Devegowda D., Dewan S.M.R., Dhane A.S., Dhimal M., Dhulipala V.R., Diaz M.J., Diress M., Dodangeh M., Doegah P.T., Dohare S., Doheim M.F., Dokova K.G., Dongarwar D., D’Oria M., Doshi O.P., Doshi R.P., Douiri A., Dowou R.K., Dsouza A.C., Dsouza H.L., Dsouza V.S., Duncan B.B., Duraes A.R., Dziedzic A.M., Ekholuenetale M., El Bayoumy I.F., El Sayed Zaki M., Elbarazi I., El-Dahiyat F., Elgendy I.Y., Elhadi M., El-Huneidi W., Elmonem M.A., Elmoselhi A.B., Eltaha C., Emeto T.I., Esezobor C.I., Esfandiari N., Esmaeili Z., Esposito F., Etoom M., Fabin N., Fadhil I., Fagbamigbe A.F., Fagbule O.F., Faghani S., Fahim A., Fakhradiyev I.R., Falzone L., Fareed M., Fares J., Farinha C.S.S., Faris M.A.I.E.M., Faris P.S., Farjoud Kouhanjani M., Faro A., Farrokhpour H., Fasanmi A.O., Fauk N.K., Fazeli P., Fazylov T., Feizkhah A., Fekadu G., Feng X., Fereshtehnejad S-M., Ferrara P., Ferreira N., Fetensa G., Feyisa B.R., Fischer F., Flor L.S., Foley K.M., Fonseca A.C., Foroumadi R., Foroutan B., Fortuna D., Foschi M., Franklin R.C., Fridayani N.K.Y., G S., Gaal P.A., Gaidhane A.M., Gaipov A., Galali Y., Gallus S., Gandhi A.P., Ganesan B., Gasevic D., Gautam P., Gautam R.K., Gebregergis M.W., Gebrehiwot M., Gebrekidan K.G., Getacher L., Getahun G.K., Getie M., Ghadimi D.J., Ghadirian F., Ghaffari Jolfayi A., Ghafourifard M., Ghasemi M-R., Ghasemzadeh A., Ghazy R.M., Gholami E., Gholami Z., Ghozy S., Giannoni Luza S., Gilani J.A., Gill T.K., Gillum R.F., Gindaba E.Z., Gnedovskaya E.V., Goel A., Goldust M., Golechha M., Goleij P., Golinelli D., Gona P.N., Gorini G., Goulart A.C., Goulart B.N.G., Gouravani M., Grivna M., Grosso G., Grover A., Guan S-Y., Guarducci G., Guha A., Guicciardi S., Gulati S., Gunawardane D.A., Guo C., Guo Z., Gupta A.K., Gupta B., Gupta M., Gupta R., Gupta R.D., Gupta R., Gupta S., Habibzadeh F., Hadi N.R., Haghani Dogahe M., Haghi-Aminjan H., Haghmorad D., Haj-Mirzaian A., Halimi A., Hamdy N.M., Hamidi S., Hamilton E.B., Hanif A., Hanifi N., Hankey G.J., Hannan M.A., Haq Z.A., Hargono A., Harlianto N.I., Haro J.M., Has E.M.M., Hasaballah A.I., Hasan I., Hasnain M.S., Hassan I., Hassan Zadeh Tabatabaei M.S., Haubold J., Havmoeller R.J., Hay S.I., Hbid Y., Hebert J.J., Hegazi O.E., Heidari M., Hemmati M., Heyi D.Z., Hezam K., Hiraike Y., Hoan N.Q., Holla R., Horita N., Hossain M.M., Hosseinzadeh H., Hosseinzadeh M., Hosseinzadeh Adli A., Hostiuc M., Hostiuc S., Hu B., Hu C., Huang J., Humayun A., Hussain S., Huy L.D., Huynh H-H., Hwang B-F., Ibitoye S.E., Ikeda N., Ikiroma A., Ilesanmi O.S., Ilic I.M., Ilic M.D., Imam M.T., Immurana M., Inbaraj L.R., Iqhrammullah M., Iradukunda A., Irham L.M., Islam M.R., Ismail F., Ismail N.E., Iso H., Isola G., Itumalla R., Iwagami M., Iwu C.D.C.D., J V., Jaafari J., Jacob L., Jafarzadeh A., Jahrami H., Jain A., Jain N., Jairoun A.A., Jaiswal A., Jakovljevic M., Jalilzadeh Yengejeh R., Janakiraman B., Jatau A.I., Jayapal S.K., Jayaram S., Jee S.H., Jeganathan J., Jegnie M., Jema A.T., Jeswani B.M., Jeyakumar A., Jha A.K., Jha R.P., Ji Z., Jiang H., Jin S., Jin Y., Jokar M., Jonas J.B., Joo T., Jose J., Joseph N., Joshua C.E., Joukar F., Jozwiak J.J., Jürisson M., Kabir A., Kabir M.A., Kabir Z., Kadashetti V., Kalani R., Kalra S., Kamal V.K., Kamireddy A., Kan H., Kanaan M., Kandel H., Kanmodi K.K., Kantar R.S., Kapoor N., Karakasis P., Karaye I.M., Karch A., Karimi H., Karimi S.E., Karimi Y., Karimi Behnagh A., Karki P., Kasraei H., Kauppila J.H., Kaur H., Kaydi N., Kayode G.A., Kazemi F., Kazemian S., Kesse-Guyot E., Khader Y.S., Khafaie M.A., Khaing I.K., Khajuria H., Khalaji A., Khalid N., Khalil A.A., Khan A., Khan F., Khan M.N., Khan M., Khan M.J., Khan M.A.B., Khan Y.H., Khanmohammadi S., Khatab K., Khatatbeh H., Khatatbeh M.M., Khateri S., Khatib M.N., Khayamzadeh M., Khayat Kashani H.R., Khidri F.F., Khokhar M., Khosla A.A., Khosravi M., Khubchandani J., Kian S., Kim K., Kim M.S., Kim Y.J., Kimokoti R.W., Kisa A., Kisa S., Kolahi A-A., Koly K.N., Kompani F., Kondlahalli S.K.M.M., Korja M., Korshunov V.A., Korzh O., Kosen S., Kostev K., Kothari N., Kotnis A.L., Koulmane Laxminarayana S.L., Krishan K., Krishna V., Krishnamoorthy V., Krishnan B., Kruja J., Kuate Defo B., Kucuk Bicer B., Kuddus M.A., Kuddus M., Kugbey N., Kulimbet M., Kulkarni V., Kumar A., Kumar A., Kumar D., Kumar G.A., Kumar N., Kumar R., Kumaran D S., Kundu A., Kundu S., Kunutsor S.K., Kurmi O.P., Kusuma D., Kutikuppala L.V.S., Kuttikkattu A., Kytö V., La Vecchia C., Lacey B., Lahariya C., Lal D.K., Lallukka T., Lám J., Landires I., Larsson A.O., Lasrado S., Latifinaibin K., Lauriola P., Lavados P.M., Lawal B.K., Le L.K.D., Le N.H.H., Le T.T.T., Le T.D.T., Lee P.H., Lee S.W., Lee W-C., Lee Y.H., Li M-C., Li W., Li X., Li Y., Lim L-L., Lim S.S., Lin J.C., Lindholm D., Linn S., Liu G., Liu R., Liu S., Liu X., Liu X., Llanaj E., Lo C-H., Lo W.D., Lohner V., López-Gil J.F., Lorenzovici L., Lorkowski S., Lotufo P.A., Lucchetti G., Luo L., Lusk J.B., Ma Z.F., Machoy M., Madadizadeh F., Maddison R., Mahmoudi E., Mahmoudvand G., Makram O.M., Malakan Rad E., Malhotra K., Malik A.A., Malik I., Mallhi T.H., Malta D.C., Mamun A.A., Manla Y., Mansouri M.H., Mansouri P., Mansouri V., Mansournia M.A., Mantovani L.G., Manu E., Marateb H.R., Marjani A., Martini D., Martini S., Martorell M., Maryam S., Marzo R.R., Masrie A., Mathangasinghe Y., Maulik P.K., Mayeli M., Mazidi M., McKee M., McPhail S.M., Mechili E.A., Mehmood A., Mehrabani-Zeinabad K., Mekene Meto T., Meles H.N., Mendoza W., Menezes R.G., Mensah G.A., Meo S.A., Meretoja A., Meretoja T.J., Mestrovic T., Mettananda C.D.K., Miazgowski T., Michalek I.M., Micheletti Gomide Nogueira de Sá A.C., Minervini G., Minh L.H.N., Mini G.K., Mirghafourvand M., Mirica A., Mirrakhimov E.M., Mirza-Aghazadeh-Attari M., Mishra M., Misra S., Mithra P., Mohamed A.I., Mohamed J., Mohamed N.S., Mohammad A.M., Mohammadi E., Mohammadi S., Mohammadi S., Mohammadian-Hafshejani A., Mohammadzadeh I., Mohammed H., Mohammed M., Mohammed S., Mohammed S., Mokdad A.H., Molavi Vardanjani H., Molokhia M., Momani S., Monasta L., Moni M.A., Montazeri F., Moodi Ghalibaf A.A., Moosazadeh M., Moradi M., Moradi Y., Moraga P., Morawska L., Moreira R.S., Morrison S.D., Mosaddeghi Heris R., Mossialos E., Mousavi P., Msherghi A., Mubarik S., Muccioli L., Mulita A., Muniyandi M., Munjal K., Murillo-Zamora E., Muthu S., Myung W., Nabavi A., Nabhan A.F., Nafei A., Nagarajan A.J., Naghavi P., Naik G.R., Naik G., Naimzada M.D., Nair S., Nair T.S., Najdaghi S., Najmuldeen H.H.R., Nakhostin Ansari N., Nangia V., Narasimha Swamy S., Nargus S., Narimani Davani D., Nascimento B.R., Nascimento G.G., Nasrollahizadeh A., Nasrollahizadeh A., Natto Z.S., Nauman J., Navaratna S.N.K., Nayak B.P., Nayak V.C., Nazri-Panjaki A., Ndejjo R., Negoi I., Negoi R.I., Nejadghaderi S.A., Nejjari C., Nematollahi M.H., Nepal S., Newton C.R.J., Nguyen D.H., Nguyen D.H., Nguyen H.T.H., Nguyen H.Q., Nguyen N.N.Y., Nguyen P.T., Nguyen V.T., Niazi R.K., Nigatu Y.T., Nikravangolsefid N., Ningrum D.N.A., Nnaji C.A., Nnyanzi L.A., Nomura S., Noor S.T.A., Norrving B., Nawsherwan N., Noubiap J.J., Nri-Ezedi C.A., Ntaios G., Ntsekhe M., Nugen F., Nurchis M.C., Nurrika D., Nzoputam C.I., Nzoputam O.J., Oancea B., Obamiro K.O., Odetokun I.A., O’Donnell M.J., Oguta J.O., Oh I-H., Ojo-Akosile T.R., Okati-Aliabad H., Okeke S.R., Okekunle A.P., Okidi L., Okonji O.C., Oladnabi M., Olagunju A.T., Olaiya M.T., Olalusi O.V., Olasehinde T.A., Olasupo O.O., Olatubi M.I., Oliveira A.B., Oliveira G.M.M., Olorukooba A.A., Olufadewa I.I., Oluwafemi Y.D.D., Oluwatunase G.O., Omar H.A., Omar Bali A., O’Neil A.E., Ong S.K., Onwujekwe O.E., Opejin A.O., Ordak M., Ornello R., Ortega-Altamirano D.V., Ortiz A., Ortiz-Prado E., Osman W.M.S., Osuagwu U.L., Otstavnov S.S., Owolabi M.O., Oyeyemi I.T., Ozair A., P A M.P., Pacheco-Barrios K., Padron-Monedero A., Padubidri J.R., Palicz T., Palma-Alvarez R.F., Pan F., Panda-Jonas S., Pande Katare D., Pandey A., Pandey A., Pandi-Perumal S.R., Panos L.D., Pantazopoulos I., Papadopoulou P., Pardhan S., Parija P.P., Parikh R.R., Parsons N., Passera R., Patoulias D., Paudel U., Pawar S., Peden A.E., Pedersini P., Peprah P., Pereira M.O., Peres M.F.P., Perianayagam A., Perico N., Perna S., Pestell R.G., Petcu I-R., Petermann-Rocha F.E., Pham H.N., Pham H.T., Phillips M.R., Pilgrim T., Piradov M.A., Pirouzpanah S., Plotnikov E., Poddighe D., Poluru R., Popovic D.S., Postma M.J., Pourshams A., Pourtaheri N., Pradhan J., Pradhan P.M.S., Prakash V., Prasad M., Prates E.J.S., Pribadi D.R.A., Puvvula J., Qattea I., Qian G., Qiao Y., Raggi A., Raghav P.R., Raghuveer P., Rahim F., Rahim M.J., Rahimifard M., Rahimi-Movaghar V., Rahman M.M., Rahman M.H.U., Rahman M., Rahman M.A., Rahmani A.M., Rahmanian M., Rahmanian N., Rahmanian V., Rahmati R., Rahmawaty S., Raj G.M., Rajaa S., Rajendran V., Rajpoot P.L., Rajput P., Ram P., Ramadan M.M., Ramadan M., Ramanarayanan V., Ramasamy S.K., Ramazanu S., Rana J., Rana K., Rana R.K., Ranabhat C.L., Rancic N., Rane A., Ranta A., Rao M., Rao S.J., Rashedi S., Rashidi M-M., Rasouli-Saravani A., Rathish D., Rauniyar S.K., Rawaf S., Razo C., Reddy M.M.R.K., Redwan E.M.M., Rehman I.U., Remuzzi G., Rezaei N., Rezaeian M., Rezazadeh H., Rhee T.G., Riaz M.A., Ribeiro A.L.P., Rodrigues M., Rodrigues da Silva T.P.R., Rodriguez J.A.B., Roever L., Romadlon D.S., Ross A.G., Rout H.S., Roy B., Roy P., Roy S., Ruela G.A., Russo M., Rwegerera G.M., S N C., Saad A.M.A., Saber K., Saber-Ayad M.M., Sabet C.J., Sabour S., Sacco S., Saddik B.A., Sadeghi E., Saeb M.R., Saeed U., Safi S.Z.Z., Sagar R., Saghafi A., Sagoe D., Saheb Sharif-Askari F., Sahebkar A., Sahoo P.M., Sahoo S.S., Sajid M.R., Salami A.A., Salaroli L.B., Saleh M.A., Salem M.Z.Y., Salum G.A., Samadzadeh S., Samargandy S., Samodra Y.L., Samuel V.P., Samy A.M., Sanabria J., Santos I.S., Santric-Milicevic M.M., Sarasmita M.A., Saravanan A., Sarikhani Y., Sarode G.S., Sarode S.C., Satpathy M., Sattouf Z., Saya G.K., Sayeed M.A., Sayyah M., Scarmeas N., Schaarschmidt B.M., Schlaich M.P., Schmidt M.I., Schneider I.J.C., Schuermans A., Schumacher A.E., Schutte A.E., Schwebel D.C., Selvaraj S., Sen P., Senapati S., Senthilkumaran S., Sergindo M.T., Sethi Y., Seylani A., Shafie M., Shah P.A., Shahabi S., Shahbandi A., Shahid S., Shahsavari H.R., Shahwan M.J., Shaikh M.A., Shalash A.S., Shamim M.A., Shams-Beyranvand M., Shamsi A., Shamsutdinova A., Shanawaz M., Shannawaz M., Sharath M., Sharifan A., Sharifi A., Sharifi-Rad J., Sharma A., Sharma M., Sharma S., Sharma U., Sharma V., Sheikhi R.A., Shetty A., Shetty M., Shetty P.K., Shiferaw D., Shigematsu M., Shimels T., Shin M-J., Shiri R., Shittu A., Shitu A.O., Shiue I., Shorofi S.A., Shrestha S., Shuval K., Si Y., Siddig E.E., Sikdar M., Silva J.P., Silva L.M.L.R., Singh A., Singh B., Singh G., Singh H., Singh J.A., Singh K., Singh N.P., Singh P., Singh P., Sipilä J.O.T., Sivakumar S., Skryabin V.Y., Skryabina A.A., Sleet D.A., Sobia F., Socea B., Sohag A.A.M., Solanki R., Solanki S., Solomon Y., Song Y., Soraneh S., Sorensen R.J.D., Sotoudeh H., Soyiri I.N., Spartalis M., Sreeramareddy C.T., Srinivasamurthy S.K., Stachteas P., Stafford L.K., Stark B.A., Starodubova A.V., Subedi N., Subramaniyan V., Suleman M., Sultana A., Sun Z., Sundström J., Suresh V., Susanty S., Swain C.K., Szarpak L., T y S.S., Tabaee Damavandi P., Tabarés-Seisdedos R., Tabatabaei S.M., Tabatabai S., Tabche C., Tabish M., Tadakamadla J., Tadakamadla S.K., Taheri A., Taiba J., Talaat I.M., Talukder A., Tampa M., Tamuzi J.L., Tan K-K., Tang H., Tanwar M., Tarigan I.U., Tarkang E.E., Tat N.Y., Tavangar S.M., Tehrani-Banihashemi A., Teimoori M., Temsah M-H., Temsah R.M.H., Teramoto M., Tesfamariam W.B., Tesfaye Gta E.G., Thakur R., Thangaraju P., Thapa R., Thapar R., Thayakaran R., Thirunavukkarasu S., Thomas J., Thomas N.K.K., Thrift A.G., Tian J., Tichopad A., Ticoalu J.H.V., Tiruneh C., Tiwari K., Tiyuri A., Tonelli M., Topor-Madry R., Tovani-Palone M.R., Trabelsi K., Tran N.H., Tran T.H., Tran Minh Duc N., Trico D., Tromans S.J., Truyen T.T.T.T., Tsai D.H-T., Tsatsakis A., Tsermpini E.E., Turuse E.A.A., Tyrovolas S., Udoakang A.J., Udoh A., Ullah A., Ullah S., Umair M., Umar M., Unim B., Unnikrishnan B., Urso D., Usman J.S., Vacante M., Vahabi S.M., Vahdati S., Vaithinathan A.G., Vakili O., Valizadeh R., Van den Eynde J., Varga O., Varthya S.B., Vasankari T.J., Vellingiri B., Venketasubramanian N., Verma M., Veroux M., Verras G-I., Vervoort D., Villafañe J.H., Villani S., Vinayak M., Viskadourou M., Volovat S.R., Volovici V., Wafa H.A., Waheed Y., Wahood W., Wang C., Wang F., Wang S., Wang S., Wang Y., Wang Y-P., Wanjau M.N., Waqas M., Wassie E.G., Wassie G.T., Wei Z., Weintraub R.G., Weldetinsaa H.L., Wickramasinghe D.P., Wickramasinghe N.D., Wijeratne T., Willeit P., Wolfe C.D.A., Wong Y.J., Wongsin U., Wu C., Wu F., Wu Y.J., Wu Z., Xiao H., Xu S., Xu X., Yamagishi K., Yang D., Yano Y., Yarahmadi A., Yaribeygi H., Yasufuku Y., Yatsuya H., Yazdanpanah F., Yazdanpanah M.H., Ye P., Yesodharan R., Yezli S., Yi S., Yi X., Yin D., Yon D.K., Yonemoto N., Yu C., Yu E.A., Yun K., Yusuf H., Zadey S., Zafari N., Zaman B.A., Zaman S.B., Zanghì A., Zare I., Zarimeidani F., Zarrintan A., Zastrozhin M., Zemedikun D., Zeng Y., Zhang B., Zhang H., Zhang L., Zhang Y., Zhang Z., Zhao H., Zhong C.C., Zhou S.C., Zhu B., Zhu L., Zhumagaliuly A., Ziafati M., Zielińska M., Zikarg Y.T., Zoghi G., Zyoud S.H., Zyoud S.H., Johnson C.O., Roth G.A., Nair B.S., Rautalin I., Bhati A., Bisignano C., Vos T., Murray C.J.L., GBD 2021 Stroke Risk Factor Collaborators (2024). Global, regional, and national burden of stroke and its risk factors, 1990–2021: A systematic analysis for the Global Burden of Disease Study 2021.. Lancet Neurol..

[5] Song P., Fang Z., Wang H., Cai Y., Rahimi K., Zhu Y., Fowkes F.G.R., Fowkes F.J.I., Rudan I. (2020). Global and regional prevalence, burden, and risk factors for carotid atherosclerosis: a systematic review, meta-analysis, and modelling study.. Lancet Glob. Health.

[6] Bir S.C., Kelley R.E. (2022). Carotid atherosclerotic disease.. Brain Circ..

[7] Nezu T., Hosomi N. (2020). Usefulness of Carotid Ultrasonography for Risk Stratification of Cerebral and Cardiovascular Disease.. J. Atheroscler. Thromb..

[8] Saba L., Brinjikji W., Spence J.D., Wintermark M., Castillo M., de Borst G.J., Yang Q., Yuan C., Buckler A., Edjlali M., Saam T., Saloner D., Lal B.K., Capodanno D., Sun J., Balu N., Naylor R., Lugt A., Wasserman B.A., Kooi M.E., Wardlaw J., Gillard J., Lanzino G., Hedin U., Mikulis D., Gupta A., DeMarco J.K., Hess C., Goethem J.V., Hatsukami T., Rothwell P., Brown M.M., Moody A.R. (2021). Roadmap consensus on carotid artery plaque imaging and impact on therapy strategies and guidelines: An international, multispecialty, expert review and position statement.. AJNR Am. J. Neuroradiol..

[9] Howard D.P.J., Gaziano L., Rothwell P.M., Oxford Vascular Study (2021). Risk of stroke in relation to degree of asymptomatic carotid stenosis: A population-based cohort study, systematic review, and meta-analysis.. Lancet Neurol..

[10] Sanghvi D., Shrivastava M. (2022). Carotid plaque imaging.. Ann. Indian Acad. Neurol..

[11] Naylor R., Rantner B., Ancetti S., de Borst G.J., De Carlo M., Halliday A., Kakkos S.K., Markus H.S., McCabe D.J.H., Sillesen H., van den Berg J.C., Vega de Ceniga M., Venermo M.A., Vermassen F.E.G., Antoniou G.A., Bastos Goncalves F., Bjorck M., Chakfe N., Coscas R., Dias N.V., Dick F., Hinchliffe R.J., Kolh P., Koncar I.B., Lindholt J.S., Mees B.M.E., Resch T.A., Trimarchi S., Tulamo R., Twine C.P., Wanhainen A., Document Reviewers, Bellmunt-Montoya S., Bulbulia R., Darling R.C., Eckstein H.H., Giannoukas A., Koelemay M.J.W., Lindström D., Schermerhorn M., Stone D.H., ESVS Guidelines Committee (2023). Editor’s Choice – European Society for Vascular Surgery (ESVS) 2023 clinical practice guidelines on the management of atherosclerotic carotid and vertebral artery disease.. Eur. J. Vasc. Endovasc. Surg..

[12] van Velzen T.J., Kuhrij L.S., Westendorp W.F., van de Beek D., Nederkoorn P.J. (2021). Prevalence, predictors and outcome of carotid stenosis: a sub study in the Preventive Antibiotics in Stroke Study (PASS).. BMC Neurol..

[13] Cismaru G., Serban T., Tirpe A. (2021). Ultrasound methods in the evaluation of atherosclerosis: From pathophysiology to clinic.. Biomedicines.

[14] Mitchell C. (2019). Grayscale analysis of carotid plaque: An overview.. J. Am. Soc. Echocardiogr..

[15] Fernández-Alvarez V., Linares-Sánchez M., Suárez C., López F., Guntinas-Lichius O., Mäkitie A.A., Bradley P.J., Ferlito A. (2023). Novel imaging-based biomarkers for identifying carotid plaque vulnerability.. Biomolecules.

[16] Cheng Y., Wu A., Ying M., Chen X. (2023). The updated roles of new ultrasound imaging techniques in assessing carotid vulnerable plaques.. WFUMB Ultrasound Open.

[17] Della-Morte D., Dong C., Crisby M., Gardener H., Cabral D., Elkind M.S.V., Gutierrez J., Sacco R.L., Rundek T. (2022). Association of carotid plaque morphology and glycemic and lipid parameters in the northern manhattan study.. Front. Cardiovasc. Med..

[18] Płoński A., Pawlak D., Płoński A.F., Głowiński J., Madycki G., Pawlak K. (2024). Gray-scale median in patients with symptomatic and asymptomatic carotid atherosclerosis-risk factors and diagnostic potential.. Biomedicines.

[19] Mechtouff L., Rascle L., Crespy V., Canet-Soulas E., Nighoghossian N., Millon A. (2021). A narrative review of the pathophysiology of ischemic stroke in carotid plaques: A distinction *versus* a compromise between hemodynamic and embolic mechanism.. Ann. Transl. Med..

[20] Paraskevas K.I., Nicolaides A.N., Kakkos S.K. (2020). Asymptomatic carotid stenosis and risk of stroke (ACSRS) study: What have we learned from it?. Ann. Transl. Med..

[21] Zhao L., Zhao H., Xu Y., Zhang A., Zhang J., Tian C. (2021). Plaque length predicts the incidence of microembolic signals in acute anterior circulation stroke.. Dis. Markers.

[22] Saba L., Scicolone R., Johansson E., Nardi V., Lanzino G., Kakkos S.K., Pontone G., Annoni A.D., Paraskevas K.I., Fox A.J. (2024). Quantifying carotid stenosis: History, current applications, limitations, and potential: How imaging is changing the scenario.. Life.

[23] Školoudík D., Kešnerová P., Hrbáč T., Netuka D., Vomáčka J., Langová K., Herzig R., Belšan T. (2022). Risk factors for carotid plaque progression after optimising the risk factor treatment: Substudy results of the Atherosclerotic Plaque Characteristics Associated with a Progression Rate of the Plaque and a Risk of Stroke in Patients with the carotid Bifurcation Plaque Study (ANTIQUE).. Stroke Vasc. Neurol..

[24] Shi G., Fan Y., Fu M., Wang J., Chen F., Cui Y., Lu Y., Zhang B., Chen L. (2024). Analysis of risk factors for carotid artery plaque in asymptomatic adults.. BMC Cardiovasc. Disord..

[25] Poredos P., Gregoric I.D., Jezovnik M.K. (2020). Inflammation of carotid plaques and risk of cerebrovascular events.. Ann. Transl. Med..

[26] Hemmati H., Larijani A., Mollaei A., Ashoobi M.A., Nikkhoy A., Mozzayan P.V., Hajari M.A., Norouzi N., Anvari S., Farzin M., Jazi K. (2025). Prognostic role of carotid plaque MRI features in cerebrovascular accidents: A systematic review and meta-analysis.. J. Clin. Neurosci..

[27] Wang Y., Yao M., Zou M., Ge Z., Cai S., Hong Y., Gao L., Zhang L., Dong Y., Peng B., Wang H., Li J. (2021). Relationship between serum lipid profiles and carotid intraplaque neovascularization in a high–stroke-risk population: A cross-sectional study in China.. J. Am. Heart Assoc..

[28] Porcu M., Mannelli L., Melis M., Suri J.S., Gerosa C., Cerrone G., Defazio G., Faa G., Saba L. (2020). Carotid plaque imaging profiling in subjects with risk factors (diabetes and hypertension).. Cardiovasc. Diagn. Ther..

[29] Wu Z., Li X., Wen Q., Tao B., Qiu B., Zhang Q., Wang J. (2022). Serum LDL-C/HDL-C ratio and the risk of carotid plaques: A longitudinal study.. BMC Cardiovasc. Disord..

